# ‘We Often Forget It Was a Disaster’: Cross-Curricular Teacher Collaboration to Develop a Curriculum Unit on the Titanic Disaster

**DOI:** 10.1007/s11191-024-00540-0

**Published:** 2024-07-19

**Authors:** Wonyong Park, Neta Shaby, Rachele Newman

**Affiliations:** Southampton Education School, University of Southampton, Southampton, UK

## Abstract

There is a growing emphasis on integrating school subjects and cross disciplinary boundaries to address local and global challenges, particularly when teaching about complex and sensitive issues such as disasters. This study explores how the integration of science and history can facilitate learning about disasters through a cross-curricular teacher professional development project in England. Seven teachers (four history, three science) from state-funded secondary schools and two museum educators in Southampton, UK collaborated with university researchers over eight months to develop a curriculum unit on the Titanic disaster for Key Stage 3 pupils (aged 11–14). Through a qualitative analysis of teacher feedback, workshop recordings and artefacts, and interviews, we illustrate the teachers’ initial excitement at the prospect of cross-curricular integration and how this excitement was then tempered by practical and logistical challenges that prevented their integration ideas from materialising into the curriculum unit. Nevertheless, teachers found that the CPD helped them to see and attend to the connections across the curriculum. Teachers rediscovered Titanic as a tragic event with historical significance for local students, which needs to be taught with reverence and ethical sensitivity. Using the Titanic disaster as an example, the study points to the potential for cross-curricular integration and teacher collaboration in teaching about disasters holistically in secondary schools.

## Introduction

The relationship between science and disaster is a complex one. On the one hand, modern technologies such as satellite imagery, weather radars, seismic sensors, maritime communication systems, and earthquake engineering have enabled early warnings and effective disaster response. Moreover, forensic science plays a central role in disaster investigations by providing scientific evidence on the likely causes of the disaster. At the same time, modern science and technology often produce and exacerbate disaster risk. In *Normal Accidents*, Charles Perrow (1999) examined how technology interacts with society to create risks. He asserted that focusing solely on engineering solutions like warnings and safeguards is ineffective because complex engineering systems will always have an element of complexity and unpredictability. Ironically, attempts to improve safety often increase complexity, potentially leading to new avenues for accidents, as tragically demonstrated at Chernobyl. In recent years, there has been an increasing interest in risk in the science education community (Christensen & Fensham, 2012; Pietrocola et al., 2021; Schenk et al., 2019). The recent *PISA 2025 Science Framework* highlights the importance of issues related to natural and technological hazards in evaluating individual action and social policy based on scientific evidence (OECD, 2022).

Disasters are *events* (of varying durations), and this distinguishes them from related concepts such as risk and hazards, particularly in the context of education. Teaching about disasters involves tragic events in the past and learning lessons from them. Even the slow-onset, gradual disasters such as climate change and desertification have their own histories that involve people, places and narratives. As van Bavel et al. (2020) notes, historical disasters offer the opportunity to ‘identify distinct and divergent social and environmental patterns and trajectories’, (p. 1) and make comparisons across space and time to learn from past disasters. History learning can be especially relevant when a past disaster involves students’ own culture and community, because such factors can increase the historical significance of the disaster to the learner. History educators have emphasised that teaching history can facilitate learners’ attachment to community, which can contribute to participatory democracy (Barton & Levstik, 2004). Past disasters in the community with historical significance can provide a rich environment for learning science in a personally and socially relevant context.

In science education literature, disasters have been most often framed as socioscientific issues (Hamza et al., 2023; Ke et al., 2023; Rennie et al., 2018; Sadler & Zeidler, 2003) that ‘involve the deliberate use of scientific topics that require students to engage in dialogue, discussion, and debate’ (Zeidler & Nichols, 2009). It has been argued that learning about socioscientific issues such as disasters require not only the acquisition of relevant scientific knowledge and processes but also an understanding of moral, ethical, emotional and political issues (Sadler & Zeidler, 2003). At the same time, teaching socioscientific issues presents science teachers with complex challenges. These obstacles encompass various aspects, including constraints on class time (Lee et al., 2006), a lack of instructional resources and materials (Ekborg et al., 2013), and the need to motivate students’ active participation (Ekborg et al., 2013). These characteristics of socioscientific issues require teachers a set of instructional skills, such as skills to facilitate perspective taking and empathy (Kahn & Zeidler, 2016). In this view, disasters can be particularly challenging topics for science teachers to address, due to the sensitivity that arises from the loss of lives and the damages that disasters often cause. Levinson and Turner (2001) and Park (2020) each suggested that cross-curricular integration and collaboration with humanities and social studies teachers, who regularly teach ethical and social issues, can be a useful way to reduce barriers to teaching sensitive topics such as disasters for science teachers.

Against this background, this article explores how the integration of science and history can facilitate learning about disasters through an exploratory case study of a cross-subject teacher continuous professional development (CPD) project in England. Seven state-funded secondary school teachers (four history, three science) and two museum educators based in Southampton participated in three CPD workshops over three months and developed cross-curricular teaching materials about the Titanic disaster collaboratively. Using various data sources collected throughout the project, including teacher feedback, interviews, and workshop recordings and artefacts, we sought to understand what cross-curricular teacher collaboration can offer for teaching about disasters in a more relevant and sensitive way. The research question that guided this qualitative project was: What are teachers' experiences of a cross-curricular CPD programme to develop a curriculum unit on the Titanic disaster?

## Literature Review

### Disaster as a Cross-Curricular Topic for Science Education

In the ‘risk society’ characterised by the emergence and dominance of risks as a central feature (Beck, 1992), technological disasters such as transport accidents, infrastructure collapses, and chemical and nuclear accidents have become regular events rather than exceptional ones. These disasters have been of enduring interest to science education researchers (Cross et al., 2000; Davis and Schaeffer 2019; Kuroda et al., 2020; Neumann, 2014; Neumann & Hopf, 2013; Park et al., 2024), but the focus has often been on examining individual disasters rather discretely rather than the considering the continuity, trends and patterns ‘across disasters’ (Fortun & Morgan, 2015). Sociologist of disaster Charles Perrow (2013) highlighted the importance of identifying patterns in nuclear disasters that are temporally and spatially apart (the atomic bombing of Hiroshima, Japan; the plutonium plant accident at Windscale, United Kingdom; the power plant accidents at Three Mile Islands, USA, Chernobyl, Soviet Union, and Fukushima, Japan). From the viewpoint of science education, considering these technological disasters spanning across time and space offers a unique opportunity to discuss the nature of technoscience in modern society (Park, 2020).

Although knowledge of science and technology is central to understanding disasters, it is imperative to recognise the social nature of disasters. The UN Office for Disaster Risk Reduction (UNDRR) emphasises that ‘[disaster] risk is ultimately the result of decisions that we make. We make decisions about the hazards to which we are willing to expose ourselves, we make decisions about where to build schools, factories, dams and dykes and how much to invest in disease surveillance and we make decisions about how our societies organize and care for vulnerable people and assets’ (UNDRR, n.d.). Since learning about disasters that involve loss of life, suffering and ethical dilemmas can be emotionally difficult, the role of the teachers is crucial in developing students’ understanding of disasters while minimising the ethical risks. In addition, the interdisciplinary nature of disasters that require scientific, technological and social understandings makes collaboration between different school subject as a promising approach to teaching about them holistically and sensitively (Park, 2020). Given that curriculum integration involves ‘a particular ideological stance which is at odds with the hegemonic disciplinary structure of schooling’ (Venville et al., 2002, p. 51), creating meaningful learning opportunities based on local contexts through curriculum integration can also cause challenges that come from the conflicting stances towards schooling, some of which we discuss later in this article.

### Cross-Curricular Teacher Collaboration

The benefits of interdisciplinarity and cross-curricular learning have been recognised widely in policy and research (Broggy et al., 2017; Drake & Reid, 2018; Euridyce, 2011). Integration of subjects can help organise the curriculum around issues in the real world, stimulate students’ interest in issues relevant to their lives, and broaden opportunities to apply to problem-solving new understandings that transcend disciplinary boundaries (Beane, 1997; Peppler & Wohlwend, 2018). One key condition to creating productive cross-subject learning opportunities, particularly in secondary schools, is the teachers who recognise the value of cross-subject curriculum and are equipped with the knowledge and skills to implement it. Park et al.’s (2022) study of science and religious education teachers in England, for example, found that teachers’ understanding and enactment of cross-disciplinary epistemic practices such as argumentation can vary significantly across subjects with different epistemological orientations. This points to the need for teachers of different subjects to grasp the nature, aims and structure of each other’s subjects in order to meaningfully design and implement cross-subject learning experiences.

Despite the increasing focus on cross-curricular learning and the role of teacher education, research is scarce on how an integrated curriculum can be developed and implemented through cross-subject teacher collaboration including science teachers. Back in the early 2000s, Ratcliffe et al. (2005) brought together science and humanities teachers to collaborate in teams to plan and deliver lessons about the social aspects of genetics to 14-to-16-year-old pupils in England. They found that while cross-curricular collaboration was effective in developing pupils’ holistic understanding of genetics, there was little evidence of teachers’ mutual learning from expertise sharing across disciplines, presumably because it was only one event that they collaborated for (Ratcliffe et al., 2005). A recent example of cross-curricular teacher collaboration is the Oxford Argumentation in Religion and Science project, where 14 pairs of science teachers and religious education teachers in England participated in an 18-month professional development to develop their skills in teaching argumentation to Key Stage 3 students. The researchers found that, by collaborating with teachers of another subject, teachers felt that their pedagogical repertoires were expanded and broadened, and such an experience inspired them to pursue further pedagogical innovations through cross-subject collaboration (Chan & Erduran, 2023). It has been suggested that, in addition to the general requirements for effective CPD, cross-subject CPD should provide a space for teachers to engage in dialogues with other school subjects’ aims, curriculum and pedagogy, and recognise the value of cross-curricular learning (Park et al., 2022).

### Integration of Science and History

In seeking ways to integrate science and history, a useful starting point would be to consider what are the commonalities and divergences between science and history as academic disciplines and as school subjects. Although the nature of scientific knowledge and enquiry has drawn extensive attention in science education research and policy globally (Erduran & Dagher, 2014; Lederman et al., 2002; NGSS Lead States, 2013; Olson, 2018; Park et al., 2019), discussions on how science compares to other subjects such as history have been scarce.

Compared to the situation in science, the views on the aims, methods and nature of history are more divided, and one main point of divergence is the ‘scientific’ status of history. Continental philosophers of history such as Hegel and Dilthey focused mainly on the features that distinguishes the natural sciences and the human sciences such as history. Dilthey (2002) argued that the natural sciences focus on explaining non-intentional events through causal explanations, while the human sciences rely on comprehending the *significance* of human actions. On the contrary, in the Anglo-American philosophy of history, emphasis has often been on the empirical and scientific nature of historical knowledge (Nagel, 1961; Walsh, 1960). In this tradition, the main interest rests in ‘how we know facts about the past, what constitutes a good historical explanation, whether explanations in history require general laws, and whether historical knowledge is underdetermined by available historical evidence’ (Little, 2020). As such, the Anglo-American philosophers of history maintained that empirical investigation and logical analysis of the implications of these discoveries are the sole sources of substantial knowledge about the world.

Such differing positions about the relationship between history and science open up a rich range of opportunities for integrating the two, by capitalising on both the similarities and dissimilarities. History curricula (including the national curriculum in England) tend to include learning goals derived from both the continental (e.g., historical significance and consciousness) as well as Anglo-American traditions (e.g., analysis and interpretation of empirical evidence, causation) (Barton & Levstik, 2004; Metzger & Harris, 2018). Park and Cho (2022) analysed cross-curricular lesson plans generated by secondary teacher teams to investigate to what extent and how science and history can be meaningfully integrated and generate cross-curricular learning opportunities. They found that most of these opportunities were concentrated on historical analytical skills that include a grasp of ‘how historical research is conducted by asking questions, collecting resources and artifacts, and analysing them to construct knowledge about the past’ (Park & Cho, 2022, p. 460). While this is unsurprising given the similarity with the process of scientific enquiry, they called for more research that explores other avenues for the two subjects to cross-fertilise in specific historical contexts (e.g., identity and moral development). In this study, we discussed the similarities and differences between the two subjects with the teachers during the first CPD workshop as a starting point for a cross-subject collaboration and integration.

### Integration of Formal and Informal Learning

Over time, there has been increasing recognition of the significance of incorporating informal learning settings like museums into the formal education system (National Research Council, 2009). Integrating informal environments, such as museums, into the formal curriculum is essential. Informal environments offer a break from traditional classroom settings, making learning more engaging and enjoyable (Lavie Alon & Tal, 2015). Students often respond positively to such learning environments, which can stimulate curiosity and foster a deeper understanding of concepts (Falk & Dierking, 1997).

Informal environments offer real-life experiences that can help bridge the gap between theoretical knowledge and practical application. Integrating informal places into the curriculum allows students to see how the concepts they learn in the classroom are relevant and applicable in various contexts, encouraging them to grasp the material more effectively (Kisiel, 2014). This can also lead to a more holistic learning experience, students gain a broader perspective on the subject matter by interacting with different environments, cultures, and scenarios. This can lead to a deeper understanding of the subject’s complexities (Falk & Dierking, 1992; Garner et al., 2016). In the context of disasters, the role of memorials and museums in analysing the disaster, building long-term collective memory and informing the public has been recognised (Knowles, 2022). Langan and Goulding (2023) worked with the 9/11 Memorial & Museum in New York City to provide place-based professional development for teachers. They found that museum-based professional development could provide valuable learning opportunities about disasters for teachers, and that teachers could translate their personal place-based experiences into their lesson plans and pedagogy after returning to their school districts.

Museums are valuable cultural resources and distinct educational settings (DeWitt & Storksdieck, 2008). These institutions offer hands-on experiences that diverges from the norm of everyday life (National Research Council, 2009), alongside extensive assortments of physical and conceptual objects, along with artifacts of considerable cultural significance (Paris & Hapgood, 2002). The learning that occurs within the museum context encompasses a variety of experiential and informational sources, collectively contributing to the individual’s process of constructing knowledge (Bamberger & Tal, 2008). Nonetheless, incorporating informal places into the formal curriculum requires thoughtful planning and alignment with learning objectives. When done well, this integration can enrich the educational experience and better equip students for the challenges they will face in their personal and professional lives (DeWitt et al., 2008; Falk & Dierking, 1992; National Research Council, 2009).

## Research Context

### Southampton and Titanic

The history of the Titanic disaster is woven into the heritage of Southampton where this study was situated. The offices of the White Star Line still stand and Dock Gate 4, from where Titanic launched, is still a busy industrial berth. There are memorials placed around the city including to the firefighters, stewards and crew and to the musicians who perished aboard the ship. A museum dedicated to Southampton’s maritime past holds a permanent Titanic exhibition with a clear focus on the people of Southampton whose lives were so impacted by the disaster. Of the crew, 724 lived within the Southampton area and only 175 of these survived. The significance of the disaster for the city of Southampton is clear. Southampton’s Old Cemetery has 44 headstones that bear the names of residents who died from the disaster. However, the disaster's impact was not just local. Southampton’s status as a bustling port ensured a multi-national city demography. A large transient population of sailors regularly boarded vessels at Southampton. Titanic also collected passengers from Cherbourg and Queenstown (currently Cobh), where there is also a memorial to the disaster. There were in fact 28 nationalities aboard Titanic, drawn from five continents. This was a disaster with true international scope.

The events of the Titanic disaster and resulting official British and American Inquiries (https://www.titanicinquiry.org/) allow an opportunity to reflect on a range of issues of interest to history and science educators. When studying Titanic, history educators might choose to approach this through the disciplinary concept of causation. This might result in questions such as ‘What was the main reason for the sinking of Titanic?’ and subsequent activity around establishing causal chains and weighing evidence to make a reasoned judgement. Opportunities arise here for the exploration of scientific principles such as buoyancy, density and displacement. However, from a history education perspective, a question such as this risks straying into the realm of simple description of events in a causal sequence without proper exploration of the ‘why’ and may lose the nuance and layered exploration of true historical study.

### Science and History Education in England

Both science and history form part of a statutory National Curriculum taught in state-maintained schools in England. The National Curriculum, since its introduction in 1988, has been revised several times in a series of top-down initiated changes. It is delivered through centralised Key Stages which span the ages ranges between 5 and 14 years old. Science is taught as a combination of physics, chemistry and biology and is deemed a core subject for mandatory study until the end of Key Stage 4 (i.e., 16 years old). History is defined as a compulsory foundation subject in early education (up to the end of Key Stage 3) and becomes an option for study at Key Stage 4. Subjects are separated into departments. The teaching of the National Curriculum is strongly influenced by high stakes assessment connected to subject specific curricular content. Schools in England are also subject to curriculum inspections by the Office for Standards in Education (Ofsted) whose inspection framework is underpinned by the English National Curricula for each separate school subject. These rigid accountability considerations remove teacher curricular agency (Sinnema et al., 2020) and may lead to teachers being reluctant to veer away from the flexibility to deliver the curriculum through an inter-disciplinary approach.

The current National Curriculum, published in 2014, represented a significant shift away from the preceding skills-based programmes of study to a content driven curricula which prioritised subject and disciplinary knowledge. It elevated the uniqueness of subject specific knowledge and arguably minimised the opportunities for interdisciplinary skills-based teaching and learning. Aston (2023) reflects that ‘the subject specific nature of curriculum making poses a challenge for curriculum development at cross-subject level’ (p. 186).

Despite this reframing, there are clear aspects of science and history National Curriculum that have commonality. Science educators are encouraged in the non-statutory curriculum guidance to provide opportunities for students to use the local environment to support their scientific investigations. History educators are required to explore local history and field studies to support student understanding of the history of where they live. The notion of ‘working scientifically’ in the science curriculum ensures that students studying science embed the key features of scientific enquiry, the review of secondary sources and the answering of ‘questions through collecting, analysing and presenting data’ (Department for Education, 2014). There are parallels here with the process of historical enquiry as presented in the National Curriculum (Department for Education, 2014) which involves the rigorous use of evidence and data to support historical claims.

## Methods

This qualitative research was designed as an exploratory case study (Yin, 2017) where we sought to understand more general problem of cross-curricular teacher collaboration on disaster education through this case. A case study approach was suitable as we wanted to gain in-depth understanding of the participants’ experiences over time. 

### Participants

Participants in this research were seven teachers: three science teachers (pseudonyms: Cindy, Amy and Margaret) and four history teachers (pseudonyms: Cole, Naomi, Melanie and Connor) from state-maintained secondary schools in Southampton, all teaching Key Stage 3 students, whose teaching experience ranged from 5 to 14 years. In addition to the teachers, two experienced museum educators at the SeaCity Museum, Lydia and Aaron, led the museum tour, joined part of the workshops, providing information about the Titanic disaster itself as well as pupils’ ideas and reactions about it during their visit. The participants were recruited through an initial teacher education partnership network of school-based subject mentors. Participants were invited to three workshops, aiming to develop a curriculum unit about Titanic. Ethical approval for the study was received from the University of Southampton’s Faculty of Social Sciences Research Ethics Committee (Approval Number 79856). Informed consent was obtained from all participants prior to their participation, and we sought consent again at the time of the interviews, which took place five to six months after the initial consent. Teachers participated in the study in their own time and were compensated for their time and contribution, taking into account their dual role as research participants in a CPD project and co-developers of a curriculum unit.

### CPD

The three workshops had two main purposes: to serve as an opportunity for teachers’ professional development and learning, and to produce a unit by bringing together the expertise of teachers and museum educators, along with theoretical and empirical knowledge about cross-curricular integration and disaster education. We adopted a collaborative professional development model where ‘nurturing learning communities within which teachers try new ideas, reflect on outcomes, and co-construct knowledge about teaching and learning in the context of authentic activity’ (Butler et al., 2004, p. 436). The intention was that the collaborative CPD would serve as a community of practice (Lave & Wenger, 1991) that provides opportunities for reflecting on their own practice and making meaningful shifts, and ‘generates energy and enthusiasm that fuels persistence with innovations’ (Butler et al., 2004, p. 438).

The workshops were designed and implemented based on general principles of effective professional development, such as (a) allowing sufficient time for teachers to assimilate and reflect on new knowledge, (b) forming a community of practice where a group of teachers work towards a shared goal with the support of external expertise (this can be people that do not work in the same school), and (c) integrating training in subject knowledge and training in general pedagogical techniques (Cordingley et al., 2005; Desimone, 2009). We incorporated these principles by (a) scheduling three 2.5-h workshops over three months to allow sufficient time for learning, interaction and reflection, (b) recruiting teachers from different departments and schools to facilitate sharing of expertise and also involving museum educators as external experts (i.e., external to the curriculum development team) with extensive knowledge of Titanic and its teaching, (c) encouraging teachers to seek cross-curricular integration while also identifying connections to the national curriculum for science and history. All three authors contributed to the workshop planning and facilitation, with the support of an undergraduate research assistant. Table 1 shows a summary of the three CPD workshops.

**Table 1:** Outline of CPD workshops

Date	Phase of CPD	Activities
February 2023	Workshop 1 (150 m)	Introductions Purpose of the project Why integrate science and history? Benefits and approaches Comparing the Key Stage 3 Science and History programmes of study Why is informal education important? Strategies for integrating informal and formal learning Guided tour of the SeaCity Museum Sharing initial ideas about the unit
May 2023	Workshop 2 (150 m)	Summary of Workshop 1 Place and community-based learning Disaster and tragedy education Developing a skeletal scheme of work for six lessons Planning workflow Wrap-up
	Individual work between workshops	Preparing the stories of six passengers on board Preparing practical activities
June 2023	Workshop 3 (150 m)	Summary of Workshop 2 Developing lesson resources Exchanging feedback Reflections on the project
July to September 2023	Collaboration after workshops	Collating teacher and student materials

Workshop 1 took place at the SeaCity museum and was divided into three parts. In the first part, the researchers introduced the rationale for developing the curriculum unit and the ideas anchoring it, which included cross-curricular integration, informal learning, place-based education and disaster education. During the second part of the workshop, participants enjoyed a guided tour of the Titanic exhibition within the SeaCity Museum. Aaron from the Museum provided insights into the artifacts and exhibits, offering additional information about the Titanic disaster. During the tour, Aaron focused on two exhibits: the first was a wall of photographs of passengers and crew on the Titanic, that appear at the entrance to the exhibition hall. Aaron highlighted the personal stories of some of the passengers and crew, underlining aspects such as demographics and social class. The second exhibit was the map of Southampton that illustrates the households in the city that were affected by the disaster. This exhibit illustrates the specific significance to Southampton. The teachers spent time exploring the map, brainstorming ideas how to incorporate it and the personal stories in the unit. In the third part, participants collaboratively engaged with Key Stage 3 science and history curricula to identify common ground between them. Working in small groups, they brainstormed ideas and captured them on sticky notes, while making connections to specific exhibits and how those can be incorporated in the lesson plans.

Workshop 2, hosted at the University campus, commenced with a brief recap of the project’s aims and the content covered in the first workshop. Participants then began developing the curriculum unit in their respective subject teams. The museum educator was available to assist with any content-related queries from the teams. Afterward, each team shared their lesson plans with the entire group to receive feedback and identify interdisciplinary areas where both subjects could intersect. After the workshop, the teachers worked individually on agreed tasks during the workshop (e.g., preparing the stories of six passengers on Titanic, and preparing practical activities).

Workshop 3 also took place at the University campus. During this session, the teams presented their final lesson plans to receive feedback. The history team teachers focused on finalising one lesson each, while the science team collaborated on refining all the lessons together. The remainder of the workshop was dedicated to perfecting all the materials, including lesson plans and slides for teachers, all of which are available in the curriculum unit portfolio. The session closed with the project team and participants sharing their reflections. After this workshop, four of the teachers continued to work asynchronously for two months to collate and refine the materials for dissemination.

### Data Collection

We used three data sources in this research. All workshops were audio and video recorded, and selections of the recordings (e.g., discussions between the teachers) were transcribed. Each participant was asked to complete a feedback survey after Workshop 1 and Workshop 2. The survey included questions about their impressions, expectations and reflections, any aspects of the project that impacted their thinking, and considerations for the collaborative curriculum development. In addition, the participants were interviewed at the end of the project. These semi-structured interviews took place online and lasted between 36 and 45 min, with the first author conducting all interviews. One participant provided a written response to the interview questions due to availability. In the interview, the teachers were asked about their experiences of the project and their reflections in general, as well as specific questions focused on cross-subject integration, place-based education, and the sensitivity of the issue. Some survey responses were followed up during these interviews. The workshop artefacts (e.g., completed worksheets and brainstorming notes) and the lesson materials developed by the teachers were also collected to complement the qualitative analysis.

### Data Analysis

The analysis followed a qualitative interpretivist approach. The three authors performed thematic analysis following Braun and Clarke's (2006) six phases of thematic analysis. In the first phase, all researchers familiarised themselves with the transcripts of the workshops, interviews and feedback, creating memos to form and record initial impressions. In the second phase, the research team engaged in initial coding and brainstorming while reading the transcripts together and developing initial themes. We took a flexible approach to coding and used both a priori codes informed by relevant theories in informal learning, disaster education, science education and history education, and inductive codes that emerged from the data. This process led to identifying four themes that are relevant to the research questions: the initial excitement, ideas for cross-curricular integration and the failure to materialise them, teachers’ professional learning, and respect and sensitivity in disaster education. In the third phase, each researcher coded a third of the data, according to the initial themes. The themes were revised as the analysis progressed. To ensure credibility, all involved in the analysis went through the process of ‘peer debriefing’, for transferability purposes we offer thick descriptions of the data (Lincoln & Guba, 1985). We also looked for both confirming and disconfirming evidence across different data sources (Erickson, 1986). The result of the analysis was reviewed by all three authors and discussed until an agreement was reached.

## Findings

### Theme 1: Teachers were Initially Excited by the Prospect of Cross-Curricular Integration to Teach about Titanic

The feedback after the first workshop and the interviews suggested that there was a clear sense of excitement at the beginning of the project. The teachers commonly expressed that they had little experience in collaborative curriculum design with teachers of subjects other than their own. They had attended workshops and professional development events with other subject teachers, which were focused on improving subject-general practices such as assessment. Yet, working with other departments for curriculum collaboration was rare, mostly because of the lack of exchange between school departments:

I think it was a really nice idea to be able to work with people from different curriculums and in the sense of sort of, I don’t know very much about the history department. You know, we’re in a very big school. And we started the year with 22 science teachers. So to sort of be able to speak to anybody in a different department is really sort of doesn’t happen very often. (Melanie, interview)

As a ‘new’ experience with the prospect of developing a cross-curricular unit, the Titanic workshops enthused teachers for the project. Cole, a Head of History in his school, had collaborated with religious education (RE) teachers in his school previously but not with science teachers. He described the project experience as ‘going outside the comfort zone’:

I’ve collaborated previously with RE teachers because there’s often that overlap with things like civil rights and the Holocaust and topics that we teach like that. So in the past I’ve worked with them on developing schemes of work that go between the two subjects. I’ve taught other subjects, so I’ve got relationships with other departments that I can chat to, but it’s the first time really where I’ve gone outside of the comfort zone for a planning perspective or curriculum design perspective. (Cole, interview)

Two particular elements of the CPD that boosted the excitement were the curriculum comparison activity and the guided tour to the museum, both during the first workshop. Connor, a history teacher, found strong crossovers between the two subjects. Attending the workshops and co-designing a curriculum with science teachers made him realise the similarity between the activities of scientists and historians:

Planning with a scientist made me realise how much potential crossover there is between subjects and how are students could benefit if all teachers were more aware of these and how it could help build schemas in their teaching. For example, there is crossover with how scientist conduct an experiment with how historians investigate the past. (Connor, workshop feedback)

This sentiment was shared by science teacher Amy, who said ‘I really enjoyed working with other teachers from other schools and from History. It was really interesting to see how our two subjects overlapped and had quite a few similarities in the way we teach’. (interview).

The guided tour of the museum served for the teachers as a rich source to gain background knowledge about Titanic from the stories and exhibits, as well as develop ideas for connecting Titanic to the curriculum of their subject. Melanie stated that ‘I also liked having time to explore the museum to think specifically about where science and history could link or support each other’ (feedback). Aaron from the museum had ‘fingertip knowledge’ and ‘helped massively with just being able to answer questions that we would have spent hours researching or Googling’ (Cole, interview). The teachers also found the visualisations, maps, multimedia resources, and technical details about Titanic that were on display in the museum useful in understanding the context of the disaster.

### Theme 2: Many Ideas were Developed for Cross-Curricular Integration, but they Did Not Materialise into the Final Curriculum Unit

As described above, the CPD helped the teachers to recognise and identify many potential avenues for cross-curricular integration between science and history to teach about a disaster. During the collaborative planning, several ideas for cross-curricular teaching were proposed and discussed. At the planning session immediately after the museum tour, Amy, a science teacher, brainstormed many areas of science that could potentially connect to Titanic based on what she observed in the museum. She suggested a range of science curriculum topics that can be highlighted in the context of Titanic, such as combustion, energy transfer and the change of states in the steam engine, and what was on board, and what the ship was made of. She also found potential relevance in the morse code, lifeboats, survival chances, and how new technology such as helicopters and life rafts that we have today might help if a similar maritime disaster happened again.

The sharing of curriculum knowledge between science and history teachers paved the way to exploring potential avenues for cross-curricular integration. The following excerpt from the second workshop is one example that shows the teachers’ efforts to plan a unit that integrates the two subjects in a balanced and interconnected way:

Naomi (H)*: Yeah, is there a way that, maybe we could, maybe something to do with interpretations. Again, I don’t know how it would work with science, but for history, we could take, it would require reading.

Melanie (S): That links nicely, because if we’re going to do why it sank, and we’re going to do history causation and look at accountability, there’s also that scientific, you know, was it the rivets, was it this, was it that.

Connor (H): If that was the claim that everyone had equal chance of survival, and then from a scientific point of view, could they then investigate it, and also historically as well, in terms of the class structure, that could be a good crossover.

Naomi (H): So could we do something like challenging the official inquiries, everybody had [equal chance of survival], well they didn’t obviously, did they? So maybe getting them to get to that point.

Amy (S): Do we know where each class was on the ship, because we could say how long did it take for each. If the lower class were at the bottom of the ship, would it have taken them longer, would they have flooded first.

Naomi (H): Like where did it hit and things like that.

(*H: History, S: Science, M: Museum)

In this conversation, history teachers Naomi, Melanie and Connor propose and elaborate historical interpretation as a potential focus for the unit, referring to specific issues such as the chance of survival and the class structure. Amy, a science teacher, then comes in and asks whether there are detailed evidence and data available to add science to the lesson. In another excerpt from the same workshop shown below, the teachers explore the possibility of ‘parallel’ lessons between science and history:

Cole (H): Would it make sense, I’ve written down six ... if we did a lesson each on those, and then try and find a parallel science equivalent. So like, causation, why did it sink, similarity and difference, looking at the different class of passenger, interpretations like you said about the review thing, change and continuity, looking at, like you said, would it be the same now, what lessons have we learnt from it, each of those lessons we’ve come up with ties in with a different second-order content, if we based it around those, the six different key skills. We could find like an equivalent skill [for science].

Amy (S): We can link something to science in each of them, even like with the interpretation, we have to look at, you know, "This is a scientific paper, like do we believe it, is it peer reviewed?". But again, we can do that with the interpretation, like is it a first-hand account, we can definitely do something like that.

Naomi (H): We can challenge it, in history that comes quite naturally, but maybe in science?

Amy (S): I mean we do have some things like that, where we have to give them like who would you believe, like this person says this is true, *kind of the same as you do in history,* you’d read a paper like this, probably peer check it, there is loads of acknowledgements, probably peer reviewed, you can have a look and see the references, but you know if it was just me saying this is my story of the Titanic, like we could compare things like that, we could definitely do some sort of interpretation as well, but I think that might be more history for that lesson, we could link it to science definitely, but it will probably be a similar lesson I think.

What can be observed in the two conversation excerpts is how the sharing of expertise between the two subjects could benefit the collaborative curriculum development process. As mentioned above, the teachers had little knowledge of the other subject’s curriculum and therefore cautious about their proposals for cross-curricular integration—as apparent from Naomi’s statement that ‘Again, I don’t know how it would work with science’—but the exchange of ideas gradually allowed the teachers to come up with ways for integration based on a communal understanding of the curricula. In the second excerpt, the connections that the teachers are exploring are at the level of what Cole refers to as ‘second-order’ contents and skills that underpins each subject, such as interpretation, learning lessons, changes and continuity, credibility and analysis.

**Fig. 1:**
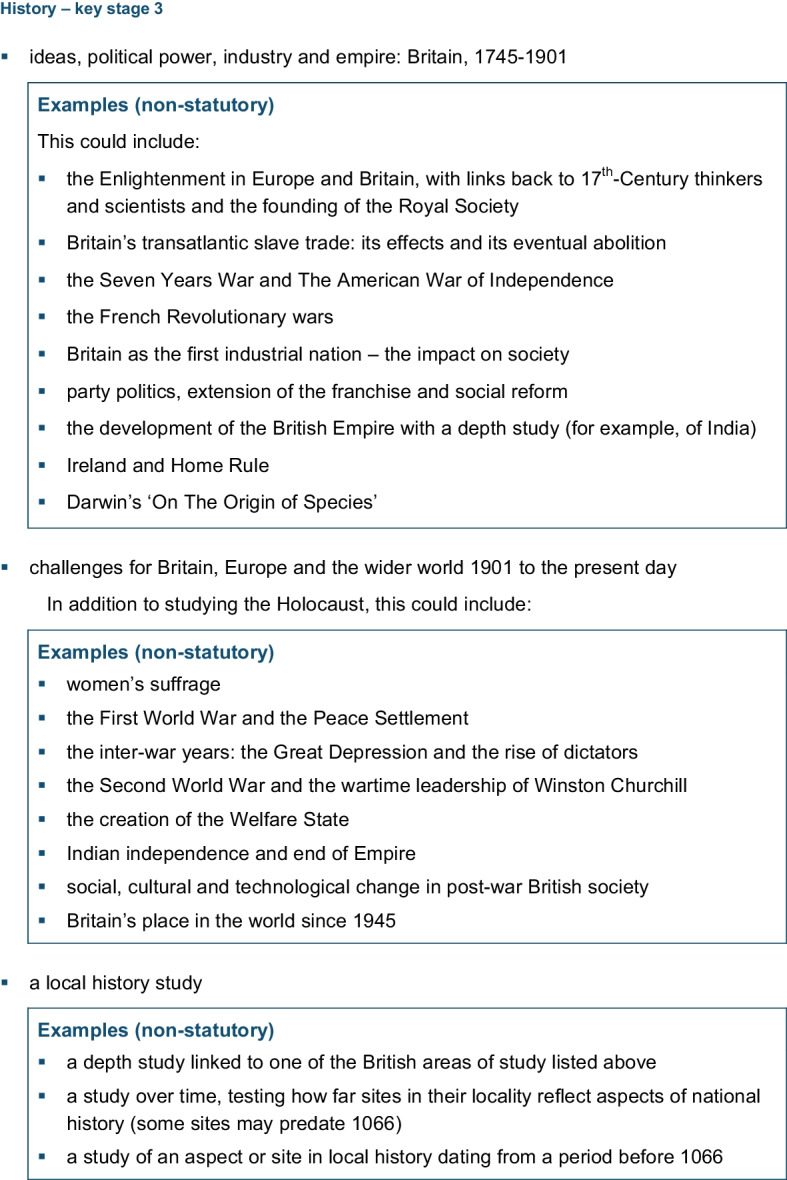
Excerpt from the Key Stage 3 history programme of study (Department for Education, 2014)

While history teachers could readily identify ways to justify the Titanic unit in their curriculum and develop the lessons accordingly, for science teachers this turned out to be a more challenging task. The main reason for this is related to how the history and science curricula were each structured. The history curriculum only provides broad topics to be covered, leaving the specific cases to illustrate the topic at the discretion of schools and teachers. The national curriculum excerpt shown in Fig. 1 includes two attainment targets that the history teachers could find connections to Titanic. The stories of the migrants aboard on Titanic can be used to exemplify the ‘challenges for Britain, Europe and the wider world 1901 to the present day’, while the close historical connection of Southampton with Titanic would make the disaster a rich ‘local history study’.

**Fig. 2:**
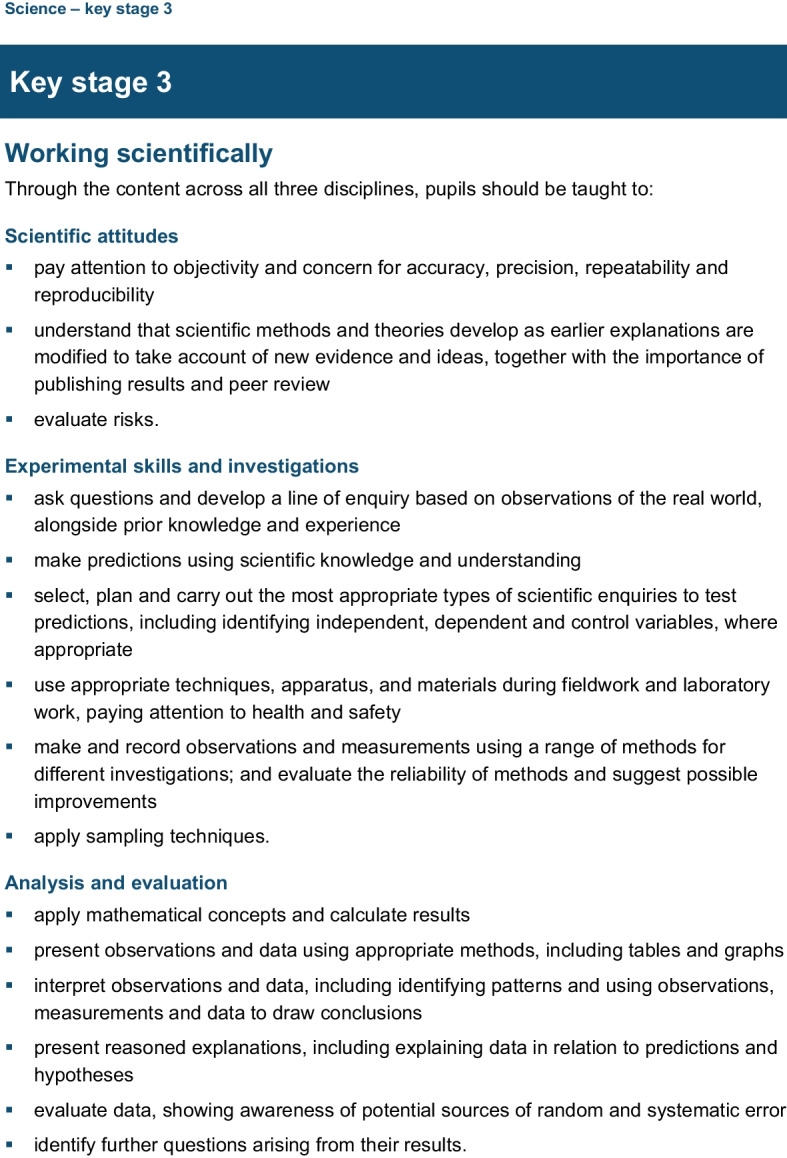
Excerpt from the Key Stage 3 science programme of study (Department for Education, 2014)

The situation for science was different. All attainment targets in the national curriculum are required by law to be taught in state-funded schools, which leaves little flexibility in how teachers organise the curriculum for teaching the content areas, that is, physics, chemistry and biology. During Workshop 2, there was a proposal made by Amy to connect Titanic to the attainment targets for ‘nutrition and digestion’ around the relationship between class, nutrition, body fat and chance of survival, but this idea was rejected when Aaron pointed out that there was no evidence that nutrition or diet had an impact on the chance of survival. Given the challenge of relating a specific disaster example to disciplinary attainment targets, a more promising approach was to find connections to attainment targets related to more generic scientific skills and attitudes, which are listed under a section called ‘working scientifically’ in the curriculum (Fig. 2).

Table 2 summarises the unit developed by teachers. The unit begins with four history lessons, each focusing on the concepts of historical significance (Why was Titanic significant to Southampton?), blame and accountability (Who was to blame for the sinking of Titanic?), impact (How far did Titanic disaster have an impact on the world?), and the disastrous nature of its sinking (Why was the sinking of Titanic a disaster?). After understanding the historical context through these four lessons, pupils attend two science-focused lessons, where they test the effectiveness of bulkheads through a practical activity, and then come up with and try ideas to make a ship more resistant to sinking. These lessons, integrated with a hands-on practical activity, were designed to support National Curriculum goals related to scientific process and enquiry skills, which are listed in the Appendix.

**Table 2:** Outline of the schemes of work

	Lesson title	Lesson content
1	Why was Titanic significant to Southampton?	**Resources:** significance table, individual experience, lesson presentation, post-it notes, interactive map **Learning objectives**: • Make inferences from individual experiences about why the Titanic was significant to Southampton • Reach a well explained judgement on why the Titanic was significant to Southampton **Outline** • Introduction to the context of Titanic within Southampton City • Exploration of broader significance criteria in historical study • Application of significance criteria to individual case studies of local people affected by the Titanic disaster • Construction of evaluative judgements on local significance
2	Why was the sinking of Titanic a disaster?	**Resources:** video clip, images of sources **Learning objectives:** • Explore and understand the popular misconception that Titanic was unsinkable • Justify why the sinking of the Titanic could be deemed a disaster **Outline:** • Examine contemporary documents and accounts related the perceived unsinkable nature of the Titanic • Explore how Titanic actually sank • Explore how something is deemed to be a disaster and how this can be applied to Titanic
3	Who was to blame for the sinking of Titanic?	**Resources:** source material, extracts from the British and American inquiries **Learning objectives**: Explore and evaluate varying aspects of culpability related to the Titanic disaster **Outline:** • Examine key individuals and responsibilities • Review individual problems that occurred and explore related culpability • Make supported evaluative judgement related to culpability • Correlate to findings of British and American Inquiries
4	How far did Titanic have an impact on the world?	**Resources**: source cards and worksheet, line of change and continuity **Learning objectives**: Make inferences from historical sources about the local and national impact of the Titanic disaster Analyse the impact on sailing and shipping by identifying changes over time **Outline:** • Explore immediate impact on Southampton and its communities • Review source material indicating wider local and national impact • Explore responses to this impact including public and policy responses
5	Watertight bulkheads	** Resources:** pre-prepared ‘bottle boats’, plasticine, balances, scissors, water troughs, tape, stopwatch ** Learning objectives:** Investigate the effectiveness of bulkheads and to write a conclusion ** Outline** • Introduction to bulkheads and their purpose • Practical—students to change the number of bulkheads on their bottle boat and time how long it takes to sink • Students to write a conclusion • Teacher information on how the bulkheads on Titanic failed
6	Keeping afloat	**Resources:** pre-prepared ‘bottle boats’, plasticine, balances, scissors, water troughs, tape, stopwatch **Learning objectives:** Investigate different ‘anti-sinking’ ideas Evaluate the effectiveness of different ‘anti-sinking’ ideas **Outline** • Recap of bulkheads • Practical: how can you stop sinking when one bulkhead is flooded • Students to write a conclusion • Video: Could Titanic have done anything differently?

Many teachers felt that the various ideas and proposals for science-history integration that emerged during the workshops were eventually not materialised into the final curriculum unit, and cross-curricular integration was only achieved to a limited extent. Although integration occurred at the unit level, each lesson was developed around attainment targets from only one of two subjects rather than both. Margaret’s comment below illustrates the team’s sentiment about how much cross-curricular integration was achieved in the final curriculum unit that they developed:

I think the lessons they do lend themselves to each other, but I don’t think they’re fully integrated to each other. I think that sort of goes back to not knowing how sort of like my school teach history and the history teachers not knowing necessarily how their school teaches science. And I think it’s like a fear of treading on somebody else’s toes if that sort of makes sense in in not wanting to sort of really dig in and deep. You know, we’ve got some really good lessons that can go alongside each other … I don’t think they quite integrate in terms of content. They’re just around the same topic at the same time then. (Margaret, interview)

### Theme 3: The CPD Helped Teachers See and Attend to Cross-Curricular Connections

Although the curriculum unit was not as integrated as the teachers had hoped, the teachers found their involvement in the cross-curricular project a constructive experience for their teaching practice. Working with teachers of another subject inspired them with ideas to borrow from science for enhancing their own lesson. Melanie said that she could use an experiment for her history lessons:

We open up new opportunities. I think that’s so true cause you know, thinking about the science lessons that the science teachers were talking about, I could do that in history, although I’m not a huge scientist. The this, the experiments they were devising, I was like, if I taught history, I could actually add that experiment on at the end, which wouldn’t be your average history lesson. (Melanie, interview)

During the interview with Connor, while talking about the importance for students to understand the *impact* of the Titanic disaster, he articulated specific ways that he thinks science can support historical understanding. He found that a scientific approach could help pupils to *visualise* the disaster and *quantify* disasters, both of which can serve to enrich historical learning:

And what I found really interesting about the Titanic project, particularly from a science point of view is again just having those conversations with science teachers and actually we can talk about it in the human aspects and that was significant to local area, but the scientists can help. I think the students can *visualise* what that disaster looked like through the experiments they were doing or help them *quantify* it in some way. And I think again that’s why I really like using individuals because I think if you just go out X amount of people died on Titanic, that number’s quite hard for them to kind of it’s just the number to them. Whereas actually when you look at those individuals and that individual stories like the guy who hung on to the sun lounger … He kind of survived in the water and they seemed stories they can connect with. I think that’s a really useful way in for students in terms of really understanding the impacts of these disasters. (Connor, interview, italics added)

Connor also said that the project made him wonder what students learn in other subjects about topics and events that are covered in history to facilitate links across the curriculum, and that he would take ‘very small practical steps’ by speaking casually to colleagues in other departments:

In planning in the future, it’s not just science, but if I’m teaching something and I can see there’s crossover, [I would] reach out to colleagues and other departments, even if it’s just a conversation about ‘This is what I’m planning to do, or this is what we’re doing at the moment. Do you cover this in any way? Have you covered this in any way, in a way I can build on or can link up with what you’re doing?’ And even if we’re not doing it at the same time, if they then know right, that’s what they have done in history in their planet can inform them. So I think even from just having a casual conversation in the staff room, that’s gonna be something that I’ll be definitely much more aware of in planning going forward. And so yeah, it was a really, yeah, thought provoking and experience but I think has also given me some just very small practical steps that I can then take into my practise which won’t require sitting down for the last time you did on the project, but we’ll still hopefully have an impact. (Connor, interview)

### Theme 4: Teachers Rediscovered Titanic as a Tragic Event with Significant Impact, which Needs to be Taught with Respect and Sensitivity

Both science and history teachers had not necessarily thought of the sinking of Titanic as a ‘disaster’ before visiting the museum to see its impact and the stories of the people involved. During the post-project interviews, most teachers stated that the project made them realise how tragic Titanic was, which many people seem to be forgetting about. Amy said that ‘at times we lost ourselves in the project and forgot that we were talking about a tragic, disastrous event as it was so long ago, and we got caught up in the excitement of how we could teach this’ (interview). Melanie said that she became more conscious of the ‘romanticised’ image of Titanic created by popular media:

So the workshops are really made me question how we present disasters, because the Titanic, I think it’s romanticised a bit thanks to the film and people don’t necessarily remember how tragic it was. And when you look into it, it was disaster, you know it was disastrous … but kind of forgetting about the human lives that were lost, I think that’s what we forget. We remember Kate Winslet and Leonardo DiCaprio, and they’ve got some responsibility for that, I think. But yes, it’s I think that’s what makes the Titanic quite unique, because it is such a horrendous disaster. But that’s not it is, and it is remembered, but not necessarily in the right way. (Melanie, interview)

The visit to the museum and subsequent discussions in the project helped them to see Titanic as a disaster that caused trauma and loss of life for many people. Rediscovering Titanic as a disaster also had educational implications. An episode from the second workshop illustrates this well, where tensions arose as participants tried to incorporate scientific, analytical and practical elements into the unit. In the following conversation, the team discusses ‘how to survive the sinking of the Titanic’ as a promising theme to link the unit to science:

Aaron (M): For science, it could be something like how to survive the sinking of the Titanic and that gives you a lot of wiggle room, so you can always go into the collisions.

Cole (H): That’s quite a good way of spinning it, because you can still include the engineering portion of it, because if we said why did some die and others didn’t that doesn’t really tie in with that, but phrasing it the way you just did, allows us to include that.

Melanie (H): Then we could go back to the idea of where everyone was.

Amy (S): You could even say like, how could your person have survived.

Connor (H): To link both history and science, could you have something like what your chances are of surviving, would people survive if the Titanic crashed today, and looking at why they didn’t survive at the time, and what lessons have been learnt in terms of moral ones from a history point of view, and technology ones from a science point of view, that could tie in there as well.

Melanie (H): It goes nicely as well because if they’re going to do a reading on their character, they could then create a hypothesis at the start of science lessons, my chances of survival are, because.

Naomi (H): I think it just depends on when it’s taught, because if you just do the moral, oh no I suppose you could still answer that, I just think the question lends itself more to ship building, design and things like that.

As the discussion proceeded, Cole brings up the sensitivity regarding the ‘how to survive’ approach in relation to the fact that Titanic was a tragedy, comparing it with the Holocaust:

Cole (H): Just to play devil’s advocate, going back to what you were saying earlier [looks at the first author] about how we teach about disasters, would how to survive the sinking of the Titanic, is that appropriate, in a sense that I wouldn’t teach how to survive the Holocaust or something like that, if we’re considering it a disaster, with a similar sort of meaning, is that an appropriate way to teach it, or are we being more [inaudible] about it because of its time, the lack of size of people dying compared to something like the Holocaust.

Naomi (H): Yeah, I agree.

Melanie (H): But the Holocaust was non-comparable.

Amy (S): And the Titanic was an accident, the Holocaust was very deliberate .

Cole (H): I’m just giving it as a discussion point, given as we were talking earlier about how we treat disasters.

This example suggests that calling the sinking of Titanic a ‘disaster’ could change how teachers should teach about it. Cole raises this issue to question if it would be ‘appropriate’ to teach about via an activity on ‘how to survive the sinking of Titanic’, drawing a comparison with the Holocaust, a tragedy that forms a core part of the history curriculum and would require a more careful treatment in pedagogy. During his interview, Cole, once again, compared Titanic with other events in history which involved violence and trauma, such as the Holocaust, slavery and wars. He said that the workshop discussions made him realise the problems and challenges with using ‘fun activities where we fish people out of the water’ to teach about events like the sinking of Titanic, which would be an insensitive and inappropriate approach:

In history teaching, we have a little bit of a disconnect with certain things that we treat as very sacred and exactly like, you know, not necessarily just disasters, but even things like the Holocaust or like slavery. We treat them with a great reverence, whereas something like the Battle of Hastings, we would take the kids outside and reenact it and they’ll run around the playground with Shields, pretending that they’re Saxons and Normans and things. If you think about it, actually, huge number of people died there as well. But we don’t treat it with the same level of respect, I suppose. And that’s certainly true for me with the Titanic in the way I would have used to have taught it to how I would now teach it. I’m a lot more conscious of the disaster and I’m not, obviously no one teaches about Titanic without realising it’s a disaster, but it was almost more of, ‘Watch a couple of clips from the movie. We’ll do some fun activities where we fish people out of the water’, or something like that going on. The active learning sort of trend. (interview)

Similar to Cole, other history teachers were able to apply their teaching methods regarding past ‘disasters’ to Titanic once its significance as a tragedy became evident. Connor highlighted how the 20th century witnessed many disasters, including wars and epidemics, and emphasized to his students the importance of acknowledging that Titanic represented a real loss of lives:

I think by facing these individual experiences, it can really help students to think about that human impact. And it’s something that, you know, could have happened in their family or, could happen again in terms of both sinking and [inaudible] like that and really getting to think in those lines, and not just ‘oh, this just happened’. (interview)

Science teacher Margaret concurred with the importance of the teacher being empathetic about the tragic aspects of Titanic and talking about it ‘in the right sense’. The difference is that she considered respect and empathy are pedagogical matters that the teacher should be conscious of, rather than something ‘to be written in a scheme of work’.

The route we took to integrating science makes it harder to look at it as a tragedy, and but I’m not saying that’s necessarily a good or a bad thing. And I think that sort of comes across in your teaching rather than it having to be written in a scheme of work. It’s sort of how you approach the subject … The only way that you can get that across is by the teacher who’s delivering that session to be empathetic and to be sad about it, and to sort of talk about it in the right sense, then, if that makes sense. … Yeah, but I still think the teacher that stands in front of them, if they’re not empathetic about this subject, that they’re teaching you can’t, you don’t portray it across your classroom, do you? (interview)

Overall, the examples above demonstrate that the workshop provided mutual learning between teachers about how to incorporate science learning activities in a unit focused on a tragedy, and that both science and history teachers recognised the need for teaching with sensitivity and respect on Titanic and other disasters that involve loss of lives and social, ethical issues.

## Discussion

This research arose from the idea that approaches based on scientific thinking and evidence have a key role to play in teaching about disasters. Despite the inherent interdisciplinary nature of disasters as socioscientific issues, there is limited knowledge on the affordances of collaboration between teachers of different subjects in developing their pedagogical knowledge about disasters, creating instructional resources and supporting teachers’ professional development. Given the various reported challenges that science teachers encounter when planning and teaching lessons with socio-scientific issues such as disasters (Ekborg et al., 2013; Lee et al., 2006), we designed and implemented a CPD programme with science and history teachers to explore the potential of cross-curricular collaboration to develop lesson units to teach about disasters sensitively, respectfully and holistically. In the following, we reflect on the study findings in the light of existing literature and the different approaches embedded in the CPD design.

As noted earlier, the purpose of the project was twofold: CPD and collaborative curriculum development. The reflections from the research team and participants indicate that there was more success with the first goal than the second. As a CPD project, the workshops facilitated the sharing of expertise in subject knowledge and lesson planning between teachers from different subjects and different schools, and engaging with museum educators in a meaningful way. The teachers reported changes in the way they see the relationship between science and history and the project’s impact on their approach to teaching about Titanic and other disastrous events to pupils. Compared to earlier studies that used short-term cross-curricular collaboration and reported insignificant impact on teacher learning (Ratcliffe et al., 2005), the strong evidence of teacher learning from our study points to the benefits of mid- to long-term engagement, enhanced by partnership with a local museum, in cross-curricular CPD for professional learning of teachers. While such positive impact on teachers is coherent with other studies that utilised collaborative CPD (e.g., Butler & Schnellert, 2012; Butler et al., 2004), what distinguishes our study is that it used curriculum development as a context for teacher learning, with support from researchers and museum educators. With the shared goal of developing a curriculum unit, the teachers were able to develop knowledge of other school subjects, reflect on and question practices that they were used to.

On the other hand, many of the innovative and promising ideas that were raised during the workshops were not ultimately incorporated into the final curriculum unit, which deserves further scrutiny. One critical and organisational reason was that we had teachers from different departments *and* different schools. While this was an advantage in terms of the diversity of approaches and ideas, it also acted as a barrier by creating practical and logistical problems. As every educational activity is situated within the broader school-level educational context such as time constraints, assessment and inter-department relationships that are unique to each school, these issues can be most efficiently discussed and addressed when the setting is shared. Given these constraints, the teachers wished they worked in colleagues from the same school, which would have eased some of the collaborative development process. As Melanie pointed out in her interview, inviting teachers from the same school would allow them to ‘listen to how other people run their history departments [which is] different from school to school’. Such a reaction from the teachers confirms that the need to collaborate with teachers from the same school in curriculum design and implementation projects raised in the context of the USA by Friedrichsen et al. (2020) is still valid in the context of England. This also posed a challenge within the wider context of the research project, developing a curriculum unit that can be adapted ‘off the shelf’ by different teachers might be more complicated.

A similar, related issue was the integration of informal learning into the unit. Although the teachers found the involvement of a local museum and museum educators highly useful, from the outset, they realised the logistical challenges of taking the entire year group to the museum. Despite the benefits of integrating informal spaces into formal curriculum, the constraints faced by schools are not new and been reported repeatedly in the literature (National Research Council, 2009). The teachers’ negotiation between the educational benefits of the museum and the practical constraints led to suggestions for practical ways of incorporating informal learning experiences such as virtual museum tours and the use of loan boxes. In addition to the historical significance that the location of Southampton created for teachers and students in relation to the Titanic, the availability of local resources such as the museum created opportunities for community and place-based science learning (Semken & Freeman, 2008), which can foster pupils’ attachment to their own city. In this way, the spatial context of the project afforded a unique opportunity to integrate science and history across time. Furthermore, visiting the museum inspired teachers and offered new perspectives to approach disaster education. The History teachers embraced the ‘photographs wall’ exhibit and adapted it into the first lesson (using personal stories to illustrate the significance of the Titanic to Southampton). They also used an interactive map produced by museum (similar to the one on the museum floor) to explore which areas of the city suffered the greatest numbers of losses and what impact this may have had on local communities across the city. Science teachers used the museum educator’s expertise to design appropriate science experiments. From this perspective, although the teachers found it challenging to incorporate a physical visit to the museum, they were able to bridge the gap between formal and informal settings, by using ideas from the museum and incorporating them into the curriculum unit.

The experience of cross-curricular curriculum development and professional development reported in this article suggests that collaboration between science and history teachers for disaster education can provide a rich context for their professional learning, while more attention needs to be paid to school-level organisational and practical issues for curriculum development to be more successful and effective. Based on the findings of the study, future research can extend the potential of cross-curricular teacher collaboration to different teaching contexts and grade levels, while addressing the logistical challenges identified in our study. As our project had fewer science teachers than history teachers, the impact of the composition of the collaboration on the ways in which teachers work together may be a fruitful area of research. Finally, given the increasing attention within the science education community to local and global issues involving violent and catastrophic events, whether caused by natural (e.g., extreme weather events) or man-made (e.g., technological failures) hazards, further work on the potential benefits of cross-curricular collaboration between science teachers and humanities and social studies teachers in teaching about disasters with care, ethics, respect and sensitivity is imperative.

## Conclusions

In a world that increasingly requires individuals to have knowledge and skills that transcend disciplinary boundaries, this study explored the potential of cross-curricular collaboration to develop a socio-scientific issues curriculum based on a disaster with a historical connection to the city. The findings demonstrate that while there were challenges in developing a curriculum unit that integrates science and history, the collaboration over a few months served as a powerful tool for teachers’ professional development. It promoted knowledge sharing, challenged disciplinary boundaries, and encouraged reflection. The limitations stemming from inter-school collaboration suggest the value of intra-school collaboration for smoother curriculum development in the future. In the meantime, the integration of local museum resources into the unit highlights the unique opportunities afforded by place-based disaster education, exemplifying the benefits of informal learning resources in formal settings. Overall, this research advocates for cross-curricular approaches when teaching about socio-scientific issues such as disasters, with attention to moral sensitivity, local connection, and logistical complexities to maximise both teachers learning and the development of effective instructional materials.

## Appendix

### National Curriculum Links

The Titanic unit of work was intended to support learning objectives set out by the National Curriculum at Key Stage 3 (Department for Education, 2014).

#### Science

- Practical enquiry into the nature, processes and methods of science through different types of science enquiries that help them to answer scientific questions about the world around them
- Describe associated processes and key characteristics in common language, and… technical terminology accurately and precisely
- Ask questions and develop a line of enquiry based on observations of the real world as well as making their own predictions using scientific knowledge and understanding
- Select, plan and carry out the most appropriate types of scientific enquiries to test predictions
- Use appropriate techniques, apparatus, and materials during laboratory work, paying attention to health and safety

#### History

- Understanding historical concepts, including historical significance
- Gain historical perspectives by placing knowledge into different contexts
- Understand the connections between local, regional, national and international history, between cultural, economic, military, political, religious and social history; and between short- and long-term timescales
- Study of an aspect or site in local history dating from a period before 1066
- Pursue historically valid enquiries
- Understand how different types of historical sources are used rigorously to make historical claims and discern how and why contrasting arguments and interpretations of the past have been constructed
- Make connections and draw contrasts
- Identify significant events, make connections, draw contrasts (focusing on the concepts of change and continuity)
